# Irisin Maternal Plasma and Cord Blood Levels in Mothers with Spontaneous Preterm and Term Delivery

**DOI:** 10.1155/2018/7628957

**Published:** 2018-05-28

**Authors:** Tereza Pavlova, Filip Zlamal, Josef Tomandl, Zuzana Hodicka, Sumeet Gulati, Julie Bienertova-Vasku

**Affiliations:** ^1^Research Centre for Toxic Compounds in the Environment (RECETOX), Faculty of Science, Masaryk University, Brno, Czech Republic; ^2^Department of Pathological Physiology, Faculty of Medicine, Masaryk University, Brno, Czech Republic; ^3^Department of Biochemistry, Faculty of Medicine, Masaryk University, Brno, Czech Republic; ^4^Department of Obstetrics and Gynaecology, University Hospital Brno, Brno, Czech Republic

## Abstract

Irisin, an adipomyokine identified in 2012, has been investigated in association with common pregnancy complications, including gestational diabetes mellitus, preeclampsia, and intrauterine growth restriction. The objective of this study is to examine the potential role of irisin in preterm birth (PTB) by comparing its level between mothers with term and preterm labor. Maternal peripheral blood and cord blood samples were collected from 30 mothers who delivered prematurely and from 35 mothers who delivered at term. Irisin concentrations were measured in all samples using ELISA, and four common single nucleotide polymorphisms in the irisin gene were determined (rs16835198, rs726344, rs3480, and rs1746661). Univariable and multivariable regression modeling was applied to evaluate maternal and cord blood irisin concentrations in relation to preterm/term labor. Irisin concentration in umbilical cord blood was found to be associated with PTB in the univariable model (*p* = 0.046). On the other hand, no differences in maternal blood irisin levels between mothers with preterm and term deliveries were established. To the best of our knowledge, this is the first study determining irisin levels in term and preterm deliveries in maternal peripheral blood and umbilical cord blood. Our study shows a possible association between cord blood irisin concentration and PTB occurrence.

## 1. Introduction

### 1.1. Preterm Birth

Preterm birth (PTB), that is, delivery before 37 weeks of pregnancy, is a leading cause of neonatal morbidity and mortality. Around 15 million babies are currently born preterm every year; moreover, this number continues to rise [1]. The rate of this serious pregnancy complication ranges from 5 to 18% of live births depending on the country [2]. PTB may either be induced, in most cases due to maternal or fetal infection, or spontaneous. Spontaneous PTB occurs either with intact membranes or after preterm premature rupture of membranes (PPROM) [3]. PPROM, defined as the rupture of the amniotic sac before the onset of labor and prior to week 37 of pregnancy, causes approximately one-third of all PTB cases [4].

### 1.2. Irisin in Pregnancy Complications

Adipokines, that is, secretory proteins released from adipose tissue, typically include cytokines, hormone-like molecules, growth factors, and other inflammatory mediators. The role of adipokines has been investigated in association with both the physiology [5] and pathophysiology [6] of pregnancy. Specifically, adipokines are known to affect uterine contractility [7, 8], pregnancy outcomes [9], and fetal growth [10].

Irisin was identified in 2012 as an exercise-induced myokine which drives the conversion of white adipose tissue (WAT) into brown adipose tissue (BAT) [11]. One year later, Roca-Rivada et al. found that irisin also acts as an adipokine, since it is released especially by subcutaneous adipose tissue [12]. As irisin was suggested to improve obesity and insulin resistance [11, 13], its therapeutic potential in metabolic disease treatment has attracted extensive interest. Furthermore, irisin was investigated in association with many pregnancy complications. Since irisin was also suggested to improve glucose tolerance [11], its involvement in gestational diabetes mellitus (GDM) was initially examined [14–23]. In addition, due to the possible association between irisin and blood pressure [24], its role in preeclampsia (PE) was later also investigated [25, 26]. In a similar manner, the role of irisin in energy homeostasis [27] was investigated in studies focusing on irisin in fetal growth restriction [28–30]. Nevertheless, as far as we know, this is the first study focusing on the role of irisin in PTB and PPROM.

### 1.3. Aims of the Study

This study thus aims to (i) investigate irisin levels in maternal circulation and in umbilical cord at the time of delivery and (ii) compare these levels between mothers who delivered prematurely and those who delivered at term. In addition, (iii) we examined associations between four selected single nucleotide polymorphisms (SNPs) in the irisin gene and irisin levels in maternal and cord blood.

## 2. Material and Methods

### 2.1. Subjects

A total of 65 Central European Caucasian women were recruited for the present study at the Department of Obstetrics and Gynaecology, University Hospital Brno (Czech Republic). Specifically, a total of 30 mothers with preterm and 35 with term deliveries were enrolled. Moreover, 16 mothers from the PTB group had PPROM, while the rest (*n* = 49) delivered with intact membranes. Signed informed consent was obtained from all participants and archived. The study was approved by the Committee for Ethics of Medical Experiments on Human Subjects, Faculty of Medicine, Masaryk University (Czech Republic), in adherence to the Declaration of Helsinki guidelines. Information about maternal anamnesis and anthropometry, current and previous pregnancies, and socioeconomic status was obtained using a standardized questionnaire and summarized in Table 1.

Inclusion criteria for participants of the study were spontaneous conception, singleton pregnancy, spontaneous delivery, and live birth. The presence of bacterial infection was further determined by both vaginal swab and urinalysis.

### 2.2. Sampling

The peripheral blood samples were collected from each mother at the time of delivery or at least one week before delivery. In the case of preterm deliveries, sampling was performed prior to the initiation of corticosteroid or tocolytic treatment. Umbilical cord blood samples were collected from umbilical cord vessels immediately after childbirth.

The plasma samples were prepared by centrifugation of 5 ml of both maternal peripheral and cord blood samples (2500*g*, 10 min). Immediately after sampling, the resulting supernatant was collected into a clean tube, aliquoted and stored at −80°C until analysis. DNA was extracted from 5 ml of both maternal peripheral and cord blood samples using the standard method based on proteinase K, subsequently stored at −20°C until analysis. The samples were collected between 2012 and 2014, while the analyses were performed between 2016 and 2017.

### 2.3. Biochemical Analysis

The irisin plasma levels were determined using a commercially available sandwich enzyme-linked immunosorbent assay (ELISA) kit (Phoenix Pharmaceuticals, EK-067-29) according to the manufacturer's instructions. The minimum detectable concentration of irisin was 1.7 ng/ml, linear range 1.7–25 ng/ml, and intra- and inter-assay variations were below 10 and 15%, respectively. Samples were diluted 2-fold prior performing the assay with assay buffer and were measured in duplicate.

### 2.4. Genotyping

Four selected SNPs were genotyped using touchdown polymerase chain reaction followed by restriction fragment length polymorphism (PCR-RFLP). Primers were designated using the Primer3Plus program [31] as follows: GGCCCATTCTGAAAAACTAGG and ACACCTCAGGCAAGTTAAGT for rs16835198, CAGTGACTTCCCCTGAGCTT and CGACAGTTCTGGGAAACAGA for rs726344, GGAAGGAAGGGGCGGTCCTT and CATCTTCCATTGGTGGTCAA for rs3480, and TGGAGAGAGTTATGTAGGGGACA and CTTCCGCAGGCTTTATTCTG for rs1746661. Utilized restriction enzymes included BsmAI, Hpy188I, StyI, and HhaI, respectively.

### 2.5. Statistical Analysis

All statistical analyses were carried out using statistical software R (version 3.3.3.). Conventional values of *p* < 0.05 were considered statistically significant. Descriptive characteristics of variables are represented by mean ± standard deviation.

Genotype distributions were tested for Hardy-Weinberg equilibrium by Fisher's exact test.

Statistical independence was tested by Pearson's chi-squared test using contingency tables, and the correlation between two variables was expressed as Cramér's coefficient. The risk of PTB associated with the individual genotypes was evaluated by linear regression model. The adjusted odds ratios for the independent variables with their 95% confidence intervals were calculated. The proper adjustment was performed for maternal age, preconception BMI, smoking status, educational status, infection, and infant gender. As only a few cases of TT and AA homozygotes were identified (in rs726344 and rs1746661, resp.), the analysis was performed as A (GG versus GA + AA) and T (GG versus GT + TT) dominant models and the genotype categories were merged. In the case of rs16835198, T dominant model was used only in infants.

The normal distribution of variables was tested using normality tests (Shapiro–Wilk, Pearson's, Anderson–Darling, etc.). In cases of skewed variables, logarithmic and square root transformation was performed and normal distribution was tested again. Variables or transformed variables with normal distribution were compared between case and control groups using parametric tests (*t*-test, Welch's test). Otherwise, nonparametric tests were used (Mann–Whitney, Kolmogorov–Smirnov).

Univariable and multivariable linear regression models were used to investigate maternal and cord blood irisin concentrations in relation to other variables. In these models, maternal/cord blood irisin levels constituted the dependent variable while all other variables were considered independent. In the case of categorical variables the reference category was selected and compared with the other one or two categories. Multivariable linear regression model utilized the following additional independent variables: PTB status, maternal age, preconception BMI, gestational age, primiparity, smoking status, educational status, infection, genotypes, and infant gender. Missing values were imputed using Multiple Imputation by Chained Equations method, and the models were built using imputed data [32].

## 3. Results

Baseline anthropometric characteristics of mothers and infants are summarized in Table 1. As expected, maternal weight gain during pregnancy and infant birth weight and length were significantly lower in mothers with preterm compared to term deliveries.

### 3.1. Genetic Analysis of Investigated SNPs

Four common polymorphisms were identified, all in the noncoding region of the irisin gene. Both maternal and fetal genotype frequencies of all selected SNPs were in Hardy-Weinberg equilibrium, except for SNP rs3480 in maternal samples. A linkage disequilibrium (LD) was determined between all SNPs in both maternal and cord blood (*p* < 0.001 for all SNP combinations, except for LD between rs726344 and rs1746661 with *p* = 0.014) (Table 2).

No association between the genotypes of investigated SNPs and their respective maternal or cord blood irisin concentration was observed. While a weak association was observed between rs726344 in mothers and the occurrence of PTB (Cramér's V = 0.27; *p* = 0.029), no association was found between the remaining SNPs and PTB incidence.

Using linear regression model, we found lower risk of PTB for mothers carrying GA + AA genotypes compared to mothers with GG genotype in rs726344 (adjusted OR = 0.06, 95% CI: 0.01; 0.62, *p* = 0.018). With the same model, we found the higher risk of PTB for mothers carrying TT compared to the mothers with GG genotype in rs16835198 (adjusted OR = 24.94, 95% CI: 1.79; 347.92, *p* = 0.017). For rs3480, we found lower risk of PTB for mothers carrying GG compared to AA (adjusted OR = 0.06, 95% CI: 0.01; 0.64), *p* = 0.019). In rs1746661, we found no association between PTB risk and genotype.

### 3.2. Maternal and Cord Blood Irisin Levels in Term and Preterm Deliveries

**Figure 1:**
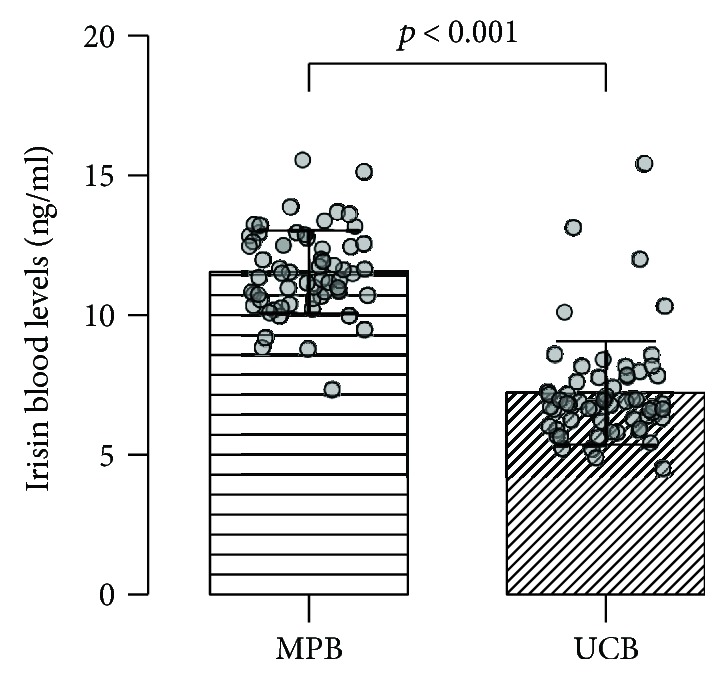
Comparison of irisin levels between maternal peripheral blood and umbilical cord blood. Individual points represent irisin concentrations in samples of maternal peripheral blood (MPB) and umbilical cord blood (UCB). Irisin concentrations are presented as mean ± standard deviation.

No significant differences in maternal or cord blood irisin levels between mothers with preterm and term deliveries were established using either the *t*-test or the Mann–Whitney test, respectively. Nevertheless, a trend towards decreased irisin levels in PTB (*p* = 0.067) was observed. Irisin levels were significantly higher in maternal peripheral blood compared to umbilical cord blood (11.6 ± 2.0 versus 7.2 ± 1.9, *p* < 0.001) (Figure 1). More specifically, maternal irisin was approximately 63% higher compared to cord blood irisin concentration. Maternal irisin levels appeared not to correlate with cord blood irisin. Similarly, no correlation between maternal or cord blood irisin levels and the other variables listed in Table 1 was observed.

**Figure 2:**
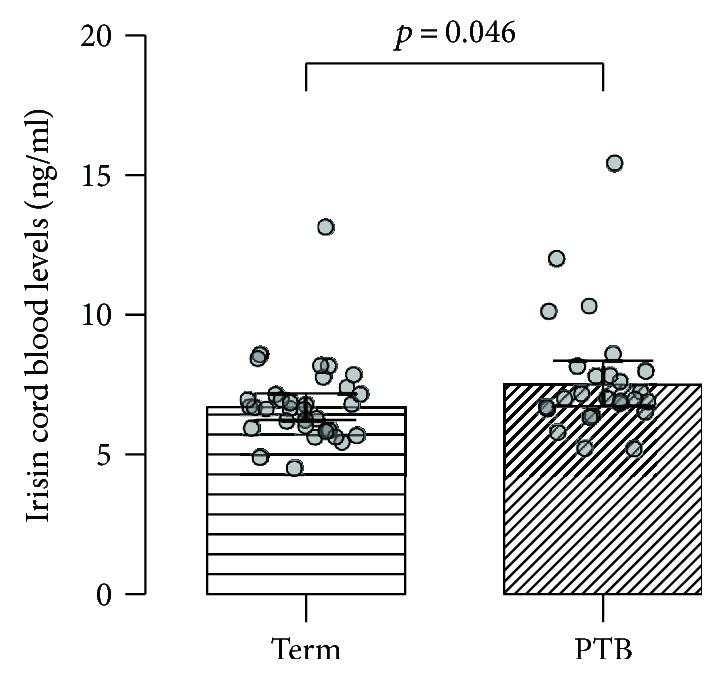
Comparison of irisin levels in umbilical cord blood between mothers with term and preterm delivery. Individual points represent irisin concentrations in cord blood samples of mothers with term and preterm (PTB) deliveries. Irisin concentrations are presented as estimated mean values with 95% confidence intervals.

The 13 variables listed in Table 3 as potential confounders in association with maternal/cord blood irisin concentrations were analyzed using univariable logistic regression. Variables used in the model included PTB status, maternal age, preconception BMI, gestational age, primiparity, smoking status, educational status, infection, genotypes, and infant gender. While according to this model maternal irisin level was not associated with any of these variables, cord blood irisin concentration was found to be associated with PTB occurrence (*p* = 0.046) (Figure 2) and smoking status (*p* = 0.025) (Table 3). In the multivariable model, maternal and cord blood irisin concentrations were investigated in relation to each of the 13 variables listed in Table 4 (i.e., identical with the above-mentioned list) and those remaining were used for purposes of adjustment. Based on this model, an association was established between maternal irisin concentration and parity (*p* = 0.046) (Table 4). The remaining variables were not significantly associated with maternal/cord blood irisin concentrations.

### 3.3. Irisin Levels in Deliveries with and without PPROM

No significant differences in maternal (12.0 ± 1.2 versus 11.6 ± 2.2 ng/ml) or cord blood (7.4 ± 1.9 versus 7.2 ± 1.9 ng/ml) irisin concentrations between mothers with and without PPROM were established.

## 4. Discussion

To the extent of our knowledge, this is the first study to analyze irisin levels in term and preterm deliveries. We demonstrated for the first time that there is no difference in maternal peripheral blood irisin levels between mothers with preterm and term deliveries. Furthermore, our study also showed a possible association between cord blood irisin concentration and PTB occurrence.

### 4.1. SNP in Irisin Gene

We found no association between the genotypes of the four investigated SNPs in the irisin gene and their respective maternal or cord blood irisin concentration. The finding is in agreement with previous studies, reporting no association between circulating irisin level and rs16835198, rs3480 [33], or rs726344 [34].

However, we observed a significant association between maternal rs726344 genotype and the occurrence of PTB, more specifically the risk of PTB lower by 94% in mothers with GA + AA compared to mothers with GG genotype in rs726344. In a recent study, Salem et al. (2018) report a significant relationship between rs726344 and PTB in an Israeli cohort. The authors reported 2.18 fold higher chance of delivering in term in mothers with GG genotype compared to AG and AA genotypes [35]. The opposite association observed in mothers in our study compared to Salem et al. could be caused by different ethnicity of both populations as well as by local geographical influences on fecundity of populations in given regions. Salem et al. investigated the rs1746661, too, and found no association with PTB as in the present study, either.

### 4.2. Irisin Association with Body Composition

The results of previous studies investigating the correlation of irisin level with metabolic parameters are controversial. While positive correlations between circulating irisin and BMI, body weight, fat mass, fat free mass, and elevated irisin level in obese patients compared to normal weight patients have been reported [34–36].

Negative correlations with anthropometric parameters (BMI, fat mass percentage, and waist to hip ratio) and decreased irisin level among obese subjects compared to lean participants have also been established [37]. Other studies have also reported an association between irisin level and insulin resistance [38, 39]. In addition, Piya et al. investigated circulating irisin in association with body composition in pregnant women and found a negative correlation of irisin level with BMI and a positive correlation with blood glucose, insulin, insulin resistance index (HOMA-IR), total cholesterol, triglycerides, and low- and high-density lipoproteins [19]. Similarly, Ebert et al. reported a positive correlation between irisin concentration and insulin, HOMA-IR, and total cholesterol in healthy pregnant women [15].

We observed an association between parity and maternal irisin concentration, specifically lower irisin levels in primiparous women after adjustment for the other variables (PTB status, maternal age, preconception BMI, gestational age, smoking status, educational status, infection, genotypes, and infant gender). Since irisin level is associated with body composition during pregnancy [15, 19] and since body composition may simultaneously be associated with parity [40, 41], we suggest that the relationship between parity and irisin concentration observed in the present study could be deduced from the different body compositions of primiparous and multiparous women. By virtue of the fact that circulating irisin is predominantly (approximately 72%) produced by muscle tissue [11, 12], and because parity influences body fat distribution and BMI [41, 42], we also assume change in muscle mass and thus an alteration in circulating irisin level.

### 4.3. Maternal Irisin Level during Pregnancy

It has further been suggested that irisin is involved in the physiology of pregnancy. During all three trimesters, irisin precursor is expressed in the placenta and the irisin serum level is higher in pregnant women compared to nonpregnant ones throughout the entire pregnancy [18, 25]. Increased maternal serum irisin during pregnancy may either be explained by placental production or it may constitute a compensatory response caused by irisin resistance during gestation [25]. With respect to the dynamics of irisin level during pregnancy, Garcés et al. reported a significant increase of approximately 16% occurring between early (weeks 11–13) and middle pregnancy (weeks 24-25) and an increase of approximately 21% between early and late pregnancy (weeks 38–40). No differences between middle and late pregnancy were detected [25]. Therefore, Garcés et al.'s results indicate a growing trend of irisin concentration during normal pregnancy.

Based on the present study, maternal irisin does not seem to be associated with the pathophysiology of PTB. On the other hand, an association between irisin level and different pregnancy complications has been reported before. Lower irisin serum concentrations were reported in mothers with GDM compared to mothers with uncomplicated pregnancies in the first trimester [16], between weeks 24–28 [21] and weeks 26–30 of pregnancy [18] as well as at term [22]. On the other hand, no significant differences between mothers with GDM and uncomplicated pregnancies in irisin levels during the second trimester [16], between weeks 24–28 [15] and at term [21], were reported. Likewise, irisin level was studied in association with PE. While Garcés et al. reported decreased maternal irisin concentration in the third trimester in preeclamptic women compared to physiological pregnancies, no differences within the group of mothers in the first and second trimester were observed [25]. In another study, no significant differences were found between PE mothers and controls before delivery [26]. Therefore, even though most studies suggest that irisin is involved in the pathophysiology of pregnancy, the role of maternal irisin in pregnancy complications remains unclear.

Our findings that irisin level in maternal peripheral blood is not associated with PTB occurrence or the other variables (except for parity) are in agreement with Garcés et al. who observed that maternal irisin level is significantly related only to insulin sensitivity during pregnancy regardless of gestational age and other variables [25]. Maternal irisin levels measured in the present study are in agreement with an existing study by Szumilewicz et al. who reported a mean irisin concentration of 14.78 ng/ml in pregnant women [43], which is consistent with our results (11.7 ± 2.0 ng/ml).

### 4.4. Cord Blood Irisin Level

Using a univariable model, we discovered a positive association between cord blood irisin concentration and PTB occurrence in the studied cohort, that is, a higher irisin level in preterm infants compared with term deliveries. Onset of labor constitutes a strong stimulus for the release of irisin into maternal and fetal circulations [44] and could increase cord blood irisin level by nearly 40% [45]. It has been suggested that increased irisin release into cord blood may be caused by temporary utero-placental ischemia during vaginal delivery, thus leading to fetal stress [44]. Similarly, fetal stress and increased cord blood irisin level secretion could occur during PTB. This mechanism could explain the positive association between cord blood irisin level and PTB occurrence observed in the present study. From another point of view, irisin improves glucose homeostasis and could compensate for metabolic changes during pregnancy [43]. Pregnancy has also been associated with increased insulin resistance [46]. Therefore, irisin could be part of a pathway maintaining glucose homeostasis during labor. Increased irisin levels detected in PTB infants in the present study suggest the impairment of glucose homeostasis which leads to PTB or, conversely, the impairment of glucose homeostasis due to PTB occurrence.

### 4.5. Irisin ELISA Kits

It is important to note that many studies based on commercial ELISA kits have evaluated irisin levels in different biological fluids; however, these studies were later called into question by Albrecht et al. (2015), demonstrating that, in addition to irisin, commercial ELISA kits also detect nonspecific cross-reacting proteins. Furthermore, the same group provided evidence against the physiological effect of irisin in the human body [47]. Nevertheless, skepticism regarding irisin was refuted and the reliability of the irisin ELISA assays was confirmed [48, 49]. Jedrychowski et al. detected and quantified irisin using mass spectrometry providing strong evidence that irisin is a true circulating protein [50]. Moreover, they offered a method that could be used as a gold standard to evaluate irisin ELISA kit validity. In terms of irisin ELISA kits, it was reported that the Aviscera irisin ELISA kit (now available from Phoenix Pharmaceuticals) correctly detected spiked irisin at physiological concentrations [34, 49]. Kits using this particular antibody were able to correctly discern both endogenous and exogenous irisin within the physiological range in humans [51, 52]. Also worth mentioning, the ELISA kit used in the present study (Phoenix Pharmaceuticals, EK-067-29) has been further validated by Western blot and verified by MALDI-TOF mass spectrometry [53].

Another limitation of the study could possibly be the partial degradation of irisin prior to analysis as no protease inhibitor was used after blood sampling (as recommended by the manufacturer of the ELISA kit (EK-067-29)). However, all the samples in our study (from the participants delivering at term as well as PTBs) were sampled using the same sampling scenario in the same facility by the same specialists so the effect observed could be explained theoretically only by huge variability in irisin degradation between the subjects which we do not presume. Moreover, Cavalier et al. (2014) reported stable irisin level during −80°C storage both with and without aprotinin for one month [54]. We therefore presume irisin should be stable after long-term storage at −80°C even without the use of protease inhibitor.

## 5. Conclusions

To the best of our knowledge, this is the first study to compare irisin levels between mothers with preterm and term deliveries. We investigated maternal and cord blood irisin levels in mothers with preterm and term deliveries and detected significantly higher irisin levels in maternal peripheral blood (11.6 ± 2.0 ng/ml) compared to cord blood (7.2 ± 1.9 ng/ml). When comparing PTB with mothers who delivered at term using a univariable model, we found an association between PTB and cord blood irisin concentration. Finally, we found no correlation between any of the selected SNPs and irisin blood concentration.

## Figures and Tables

**Table 1:** Baseline characteristics and irisin levels in study groups.

Variable		PTB	Term	All	Test	*p* Value
		*n* = 30	*n* = 35	*n* = 65		
Age	Years	28.9 ± 5.3	30.4 ± 4.7	29.7 ± 5.0	*t*-test	0.215
Height	cm	166.3 ± 6.8	169.7 ± 7.4	168.1 ± 7.3	*t*-test	0.063
Weight (preconception)	kg	64.4 ± 12.9	63.2 ± 13.1	63.7 ± 12.9	*t*-test	0.681
Weight (delivery)	kg	74.3 ± 11.6	76.2 ± 12.8	75.3 ± 12.2	*t*-test	0.513
Weight gain	kg	9.9 ± 4.9	13.0 ± 4.1	11.6 ± 4.7	*t*-test	**0.007**
BMI (preconception)	kg/m^2^	23.3 ± 4.7	21.9 ± 4.4	22.6 ± 4.6	MW	0.188
BMI (delivery)	kg/m^2^	26.9 ± 4.5	26.5 ± 4.3	26.7 ± 4.3	*t*-test	0.688
Infant birth weight	g	1887 ± 580	3357 ± 501	2678 ± 912	Welch	**<0.001**
Infant birth length	cm	43.2 ± 4.6	49.5 ± 1.8	46.6 ± 4.6	Welch	**<0.001**
Gestational age	week	32.3 ± 3.2	39.2 ± 1.0	36.0 ± 4.2	KS	**<0.001**
Maternal irisin	ng/ml	12.0 ± 2.4	11.5 ± 1.5	11.7 ± 2.0	*t*-test	0.642
Fetal irisin	ng/ml	7.7 ± 2.2	6.8 ± 1.5	7.2 ± 1.9	MW	0.067

Data are expressed as mean ± standard deviation. *p* values express the difference in variables between the PTB and term delivery group based on a selected test. *p* values in bold are statistically significant. PTB/term: mothers with preterm/term delivery; MW: Mann–Whitney test; KS: Kolmogorov–Smirnov test.

**Table 2:** Linkage disequilibrium between four irisin single nucleotide polymorphisms.

Mother	rs16835198	rs726344	rs3480	rs1746661
rs16835198	—	0.125	0.269	0.177
rs726344	0.999	—	0.091	0.006
rs3480	0.735	0.601	—	0.356
rs1746661	0.999	0.091	1.000	—
Infant	rs16835198	rs726344	rs3480	rs1746661
rs16835198	—	0.091	0.464	0.139
rs726344	0.999	—	0.115	0.052
rs3480	0.936	0.818	—	0.263
rs1746661	0.999	0.999	0.999	—

Data express the linkage disequilibrium between four irisin polymorphisms in Czech mothers and their infants. D′ values are given below the empty cells, and *r*^2^ values above the empty cells.

**Table 3:** Univariable linear regression model with maternal and cord blood irisin levels as dependent variables.

Variable	Reference category	Comparative category	Mother			Infant		
			*β*	95% CI	*p* value	*β*	95% CI	*p* value
PTB status	Term	Preterm	0.018	(−0.064; 0.099)	0.669	0.113	(0.002; 0.224)	**0.046**
Maternal age			0.002	(−0.007; 0.011)	0.647	0.004	(−0.009; 0.016)	0.557
Preconception BMI			0.000	(−0.009; 0.009)	0.965	−0.001	(−0.013; 0.011)	0.855
Gestational age			−0.002	(−0.012; 0.009)	0.764	−0.013	(−0.027; 0.001)	0.069
Primiparity	No	Yes	−0.060	(−0.145; 0.024)	0.160	−0.023	(−0.144; 0.097)	0.702
Smoking status	Nonsmoker	Smoker + former smoker	−0.037	(−0.156; 0.082)	0.538	−0.185	(−0.346; −0.024)	**0.025**
Education	Basic	Secondary	−0.004	(−0.113; 0.106)	0.948	0.062	(−0.091; 0.214)	0.420
	Basic	University	−0.004	(−0.107; 0.099)	0.942	0.048	(−0.096; 0.191)	0.509
Infection	No	Yes	0.004	(−0.091; 0.100)	0.928	0.071	(−0.060; 0.202)	0.282
Infant gender	Female	Male	−0.041	(−0.123; 0.041)	0.317	−0.055	(−0.170; 0.060)	0.341
rs16835198	GG	GT	−0.012	(−0.101; 0.078)	0.797	0.077	(−0.040; 0.194)	0.192^∗^
	GG	TT	0.096	(−0.035; 0.226)	0.147			
rs726344	GG	GA + AA	0.015	(−0.072; 0.103)	0.726	−0.046	(−0.172; 0.080)	0.467
rs3480	AA	AG	−0.069	(−0.163; 0.025)	0.148	0.042	(−0.111; 0.195)	0.583
	AA	GG	−0.030	(−0.131; 0.070)	0.547	0.013	(−0.164; 0.190)	0.883
rs1746661	GG	GT + TT	−0.001	(−0.083; 0.081)	0.979	0.035	(−0.087; 0.157)	0.569

Univariable logistic regression analysis investigates 13 variables as potential confounders in association with maternal/cord blood irisin concentrations. As only a few cases of TT and AA homozygotes were identified (in rs726344 and rs1746661, resp.), the analysis was performed as A (GG versus GA + AA) and T (GG versus GT + TT) dominant models and the genotype categories were merged. Significant results are in bold. ^∗^GG versus GT + TT (T dominant model).

**Table 4:** Multivariable linear regression model with maternal and cord blood irisin levels as dependent variables.

Variable	Reference category	Comparative category	Mother			Infant		
			*β*	95% CI	*p* value	*β*	95% CI	*p* value
PTB status	Term	Preterm	−0.026	(−0.227; 0.175)	0.796	0.212	(−0.059; 0.483)	0.122
Maternal age			−0.003	(−0.016; 0.010)	0.649	0.000	(−0.017; 0.016)	0.958
Preconception BMI			−0.001	(−0.011; 0.010)	0.915	0.000	(−0.013; 0.013)	0.955
Gestational age			0.001	(−0.022; 0.024)	0.935	0.008	(−0.022; 0.038)	0.591
Primiparity	No	Yes	−0.130	(−0.257; −0.002)	**0.046**	−0.030	(−0.195; 0.136)	0.718
Smoking status	Nonsmoker	Smoker + former smoker	0.017	(−0.161; 0.194)	0.852	−0.176	(−0.371; 0.019)	0.076
Education status	Primary	Secondary	−0.024	(−0.158; 0.110)	0.719	0.067	(−0.105; 0.238)	0.437
		University	0.045	(−0.101; 0.191)	0.536	0.083	(−0.098; 0.263)	0.361
Infection	No	Yes	0.001	(−0.121; 0.124)	0.982	0.055	(−0.103; 0.214)	0.485
Infant gender	Women	Men	−0.063	(−0.172; 0.046)	0.251	−0.012	(−0.158; 0.135)	0.873
rs16835198	GG	GT	0.032	(−0.095; 0.159)	0.613	0.157	(−0.007; 0.321)	0.061^∗^
		TT	0.178	(−0.040; 0.396)	0.106			
rs726344	GG	GA + AA	0.035	(−0.084; 0.154)	0.555	−0.140	(−0.298; 0.019)	0.082
rs3480	AA	AG	−0.081	(−0.225; 0.064)	0.267	0.082	(−0.109; 0.273)	0.389
		GG	−0.093	(−0.282; 0.096)	0.327	0.181	(−0.104; 0.467)	0.207
rs1746661	GG	GT + TT	0.117	(−0.030; 0.264)	0.116	−0.001	(−0.154; 0.153)	0.992

In the multivariable linear regression model, maternal and cord blood irisin concentrations were investigated in relation to the 13 variables listed in the table. In the case of categorical variables, a reference category was selected and compared with one or two other categories. As only a few cases of TT and AA homozygotes were identified (in rs726344 and rs1746661, resp.), analysis was performed as A (GG versus GA + AA) and T (GG versus GT + TT) dominant models and the genotype categories were merged. Significant results are in bold. ^∗^GG versus GT + TT (T dominant model).

## Data Availability

The authors declare that the data supporting the findings of this study are available within the article or are available from the corresponding author upon reasonable request.
